# Sex and gender differences in post-traumatic stress disorder: an update

**DOI:** 10.1080/20008198.2017.1351204

**Published:** 2017-09-29

**Authors:** Miranda Olff

**Affiliations:** ^a^ Department of Psychiatry, Academic Medical Center, University of Amsterdam and Arq Psychotrauma Expert Group, Diemen, The Netherlands

**Keywords:** Gender, sex, post-traumatic stress disorder (PTSD), oxytocin, neurobiology, amygdala

## Abstract

**Background**: Women have a two to three times higher risk of developing post-traumatic stress disorder (PTSD) compared to men. Several factors are involved explaining this difference (Christiansen & Hansen, 2015). Both psychosocial and biological explanations (e.g. oxytocin related) have been suggested and will be reviewed in this paper. To date, we are still behind in gender- and sex-sensitive research and reporting.

**Prevalence and type of trauma**: The lifetime prevalence of PTSD is about 10–12% in women and 5–6% in men. There are similar differences between the sexes for (comorbid) disorders such as major depression and anxiety disorders. PTSD subcluster scores have been found to be increased in women, e.g. for re-experiencing and anxious arousal (Charak et al., 2014). Men and women experience different types of trauma, both in private life and at work (e.g. police officers, Van der Meer et al., 2017), with women being exposed to more high-impact trauma (e.g. sexual trauma) than men, and at a younger age. Trauma early in life has more impact, especially when it involves type II trauma interfering with neurobiological development and personality. Traumatic stress affects different areas of the brains of boys and girls at different ages.

**Acute phase, stress-coping and psychotherapy**: In the acute phase, women generally score higher than men on acute subjective responses, e.g. threat perception, peritraumatic dissociation and known predictors of PTSD. Women handle stressful situations differently and have evolved differentially to support these different behaviours. For instance, women in stressful situations may use a tend-and-befriend response rather than the fight-or-flight response that is often assumed. Emotion-focused, defensive and palliative coping are more prevalent in women, while problem-focused coping is higher in men. Women seek more social support, the lack of it being the most consistent predictor of negative outcome of trauma. Women have been shown to benefit more from psychotherapy then men in the reduction of PTSD symptoms.

**Psychobiological reactions and effects of oxytocin**: Although only 2% of psychobiological research has been conducted in females (mainly rats), sex differences have been shown. Women appear to have a more sensitized hypothalamus–pituitary–axis than men, while men appear to have a sensitized physiological hyperarousal system. PTSD has consistently been associated with amygdala hyperactivity, ventromedial prefrontal cortex (vmPFC) hypoactivity and reduced communication (functional connectivity) between the vmPFC and amygdala, with the lower PFC control over the amygdala providing an explanation for the excessive fear response in PTSD. We hypothesized that the oxytocin system, which is associated with social support, fear and stress responses, was likely to play a sex-specific role in the stress response. In recently traumatized patients, we found that the effects of administration of oxytocin on amygdala reactivity to emotional stimuli depend on stimulus valence and sex (Frijling, 2017). In PTSD patients, we showed sex-specific routes for the effects of single oxytocin administration on the potential to diminish anxiety (fear learning) and fear expression by the amygdala: increased inhibitory control of the vmPFC over the centromedial nucleus in men and fewer excitatory dorsal anterior cingulate cortex projections to the basolateral nucleus in women. So, while our findings add to accumulating evidence that oxytocin administration could potentially enhance treatment response in PTSD, the routes in men and women differ (Frijling, 2017).

**Gender policy**: In summary, all of these sex and gender differences in brain and behaviour together may explain why PTSD is more prevalent in women than in men. Clearly, we should not simplify. There are no male or female stereotypes, but some features are more common in women and others in men. To fully understand the differences, we need more gender- and sex-sensitive research as well as reporting (e.g. see the gender policy of the European Association of Science Editors). In 2016, the *European Journal of Psychotraumatology* was the first to implement a gender policy (Olff, 2016), i.e. authors are asked to: report the sex of research subjects, justify single-sex studies, discriminate between sex and gender (mostly for human research), analyse how sex or gender impact the results, and discuss sex and gender issues when relevant. This should not only apply to the field of psychotrauma, but deserves a much broader implementation. In doing so, we hope to obtain information that will improve sex- and gender-specific approaches to helping those affected by psychotrauma.
